# Supplementary material for: A Computational Analysis of the Dynamic Roles of Talin, Dok1, and PIPKI for Integrin Activation
Source: PLoS One. 2011 Nov 16;6(11):e24808. doi: 10.1371/journal.pone.0024808 (PMC3217926; doi:10.1371/journal.pone.0024808)
Supplement: Table S1 — Mean parameter values of group 1 (high TAL∶INT, Figure 3 main text) and group 2 (high DOK∶INT, Figure 3 main text). On-rates are related to the KD value mentioned in the main text as . (PDF) [file pone.0024808.s001.pdf]

A computational analysis of the dynamic roles of talin, Dok1, and PIPKI for integrin activation - Supplementary Table

Florian Geier[1,2], Georgios Fengos[1], Dagmar Iber[1,*]

1 ETH Zürich, Department of Biosystems Science and Engineering (D-BSSE), Mattenstrasse 26, 4058 Basel, Switzerland; 2 new address: Biozentrum, Klingelbergstrasse 70, 4056 Basel, Switzerland

∗ E-mail: Corresponding dagmar.iber@bsse.ethz.ch

Parameter	Group 1	Group 2
k1on	0.68	0.4
k1off	19.749	10.467
k2on	2.353	0.950
k2off	0.706	0.285
k3aon	0.106	0.117
k3aoff	0.009	0.014
k3bon	0.018	0.055
k3boff	0.191	0.609
k5on	2.154	2.128
k5off	313.8	319.7
k6on	2.107	2.093
k6off	23.87	24.26
k7	14.89	15.09
k8	14.85	15.21
k9	14.89	15.07
k10on	3.423	2.042
k10off	0.666	0.374
k11a	0.214	0.143
k11b	0.0006	0.0015
k12	0.093	0.1
k13	0.002	0.002
k14	0.002	0.001
k15	0.0015	0.0014
k16on	2.071	2.075
k16off	0.704	0.692
k17offA	2.839	2.902
k17offB	0.03	0.033
INT_{tot}	40	40
L_{tot}	15	15
TAL_{tot}	6.98	0.68
DOK_{tot}	1.74	6.05
$PIPKI_{tot}$	0.88	1.38
SRC_{tot}	25	25

Table S1. Mean parameter values of group 1 (high TAL:INT, Figure 3 main text) and group 2 (high DOK:INT, Figure 3 main text). On-rates are related to the KD value mentioned in the main text as $kon = koff/KD$.
