# Supplementary material for: A Computational Analysis of the Dynamic Roles of Talin, Dok1, and PIPKI for Integrin Activation
Source: PLoS One. 2011 Nov 16;6(11):e24808. doi: 10.1371/journal.pone.0024808 (PMC3217926; doi:10.1371/journal.pone.0024808)
Supplement: Text S1 — A computational analysis of the dynamic roles of talin, Dok1, and PIPKI for integrin activation - Supplementary Text. (PDF) [file pone.0024808.s002.pdf]

A computational analysis of the dynamic roles of talin, Dok1, and PIPKI for integrin activation - Supplementary Text

Florian Geier[1,2], Georgios Fengos[1], Dagmar Iber[1,*]

1 ETH Zürich, Department of Biosystems Science and Engineering (D-BSSE), Mattenstrasse 26, 4058 Basel, Switzerland; 2 new address: Biozentrum, Klingelbergstrasse 70, 4056 Basel, Switzerland

∗ E-mail: Corresponding dagmar.iber@bsse.ethz.ch

Rule-based model: molecule types and total concentrations

Ligand The concentration of the ligand molecule defines the density of integrin binding sites. However, the decisive quantity in our model is the binding site density relative to the binding site affinity: L_{tot}/K_D. We have explored ranges between $10^{-10} \leq L_{tot}/K_D \leq 10^4$ and used a value of 50 for all time course simulations. Given a $K_D = 0.3\mu$M, this would correspond to ligand concentration of $L_{tot} = 15\mu$M, which is among experimentally used ligand densities [1, 2].

`L(I)`

`I`	single integrin binding site

Integrin There are typically about 5×10^5 integrins on a fibroblast [3,4] and about $1 - 30 \times 10^5$ integrins on CHO cells [5]. The surface area of CHO cells has been measured as 1000-2500 μm^2 [5]. Based on these measurements we expect a density of about 40-3000 integrins per μm^2 and we shall use 500 integrins per μm^2 as this corresponds best to both the measurements in fibroblast and CHO cells. If we assume 20 nm as the integrin-ligand binding distance then a density of 500 integrins per μm^2 corresponds to a concentration of about 40 μM.

`INT(Conf~O~C,L,S1,NPXY~U~P)`

`Conf~O~C`	Open and closed integrin conformation
`L`	ligand binding domain
`S1`	SRC/FAK beta3 cytoplasmic tail binding domain
`NPXY~U~P`	Talin/Dok binding domain [6]; gets phosphorylated by active Src [7]

Dok1

`DOK1(PTB)`

`PTB`	Integrin binding domain

Talin1

`TAL1(F2,F3~O~C)`	
`F2`	binding domain for PIP2 (membrane recruitment of talin) [8].
`F3~O~C`	PTB domain, bind integrin tails and PIPKI, is shielded in closed conformation (autoinhibition) [9–11].

Src kinase We have shown previously that the experimentally observed kinetics of Src kinase activation and deactivation can best be reconciled with a Src density of 300 per μm^2 which corresponds to a Src kinase concentration of about 25 μM and if Src kinases can interact with all integrin conformations (Felizzi and Iber, submitted).

`SRC(SH2,SH3,Y419~U~P)`

`SH2`	interaction domain with CSK
`SH3~U~P`	domain can directly interact with beta3 cytoplasmic tails

PIPKIγ

`PIPKI(SPLH,Y644~U~P)`

`Y644~U~P`	phosphorylted by SRC [9]
`SPLH`	binding domain to F3 domain of talin, Y644 regulated

Rule-based model: reaction rules, parameter values and fold changes

Rule 1a: Integrin opening and closure Integrins are allosteric proteins that exist in many conformations [12]. For simplicity we only consider the two extreme conformations, a closed, inactive conformation and an open, active conformation. The conformational equilibrium constant K_c can be derived from the observation that in the absence of ligand only about $2-3\%$ of all integrins are in the open, high affinity conformation [13]. The underlying transition rates have not been measured but a reasonable time scale is $1/10-1$ sec. `INT(Conf~C,L,NPXY~?)  <-> INT(Conf~O,L,NPXY~?)  k1on, k1off`

Rule 1b: Integrin closure Applies to all open-DOK-bound-integrins irrespective of the phosphorylation state.

```
INT(Conf~O,L,NPXY~?!1).DOK(BD!1) -> INT(Conf~C,L,NPXY~?)  + DOK(BD) k1off
```

Rule 2: Ligand binding The dissociation constants for $\alpha_V\beta_3$ integrins in the open conformation has been measured as $K_d \sim 0.3\mu$M [14,15]. As discussed in detail elsewhere [16] the affinity of integrins for surface-bound ligand is much lower than for ligand in solution [17–19]. Accordingly we use 500-fold lower affinity constant. For such a lower affinity ligand binding is limited, even at the high ligand densities that are employed in experiments. We used $k_{on} = 1\mu\mathrm{M}^{-1}$ s^{-1} as on-rate and we set the off-rate according to the dissociation constant.

```
INT(Conf~O,L) + L(I) <-> INT(Conf~O,L!1).L(I!1) k2on, k2off
```

Rule 3a: Interaction of unphosphorylated integrin and talin Talin binds to unphosphorylated integrin β tails with at a very low rate, $k3aon = 5\times10^4$ M^{-1} s^{-1}. The off-rate was measured as $k3aoff = 5\times10^{-3}$ s^{-1} so that $K_d = 0.1\mu$M [6].

```
TAL(Loc~PM,Conf~O,BD) + INT(Conf~O,NPXY~U) <->
TAL(Loc~PM,Conf~O,BD!1).INT(Conf~O,NPXY~U!1) k3aon, k3aoff
```

Rule 3b: Interaction of unphosphorylated integrin and Dok

```
DOK(Loc~PM,BD) + INT(Conf~O,NPXY~U) <->
DOK(Loc~PM,BD!1).INT(Conf~O,NPXY~U!1) k3bon, k3boff
```

Rule 4a: Interaction of phosphorylated integrin and talin Phosphorylation of the integrin beta tail reduces the affinity of binding 2-fold [20].

```
TAL(Loc~PM,Conf~O,BD) + INT(Conf~O,NPXY~P) <->
TAL(Loc~PM,Conf~O,BD!1).INT(Conf~O,NPXY~P!1) k3aon, 2*k3aoff
```

Rule 4b: Interaction of phosphorylated integrin and Dok [20]

```
DOK(Loc~PM,BD) + INT(Conf~O,NPXY~P) <->
DOK(Loc~PM,BD!1).INT(Conf~O,NPXY~P!1) k3bon, k3boff/400
```

Rule 5: Src opening and closure In the resting state Src kinases are predominantly inactive (90 − 95%) [21]. To obtain such a conformational equilibrium we set our rates for the opening and clamping to $k5on = 1s^{-1}$ and $k5off = 100s^{-1}$ respectively.

```
SRC(Conf~C,SH3,Y419~U) <-> SRC(Conf~O,SH3,Y419~U) k5on, k5off
```

Rule 6a: Src integrin interaction The concentration at which half-maximal binding of Src kinases and integrins is achieved has been determined as $EC_{50} \sim 5 - 10\mu$M [22] and accordingly we use as dissociation constant $K_d = 7.5\mu$M for the Src kinase-integrin interaction.

```
SRC(Conf~O,SH3) + INT(BD) <-> SRC(Conf~O,SH3!1).INT(BD!1) k6on, k6off
```

Rule 6b: Src PIPKI interaction

```
SRC(Conf~O,SH3) + PIPKI(Loc~PM,S1) <-> SRC(Conf~O,SH3!1).PIPKI(Loc~PM,S1!1) k6on, k6off
```

Rule 7a: integrin-dependent Src phosphorylation Activation of Src kinases is achieved by trans autophosphorylation on Tyr-418 [23] and thus requires juxtaposition of open Src kinases, presumably by binding to ligand-bound integrins [22]. We do not model juxtaposition of integrins explicitly, but require ligand bound integrins.

```
SRC(SH3!1,Y419~U).INT(BD!1,L!+) -> SRC(SH3!1,Y419~P).INT(BD!1,L!+) k7
```

Rule 7b: cross-talk dependent Src phosphorylation Inside-out signaling is simulated by setting IOFLAG=1 and $L_{tot} = 0$.

```
SRC(SH3!1,SH3,Y419~U) -> SRC(SH3!1,SH3,Y419~P) IOFLAG*k7
```

Rule 8: PIPKI phosphorylation The K_m for the optimal Src kinase substrate has been determined as $K_m = 30\mu$M, and accordingly we use $k_{cat} = 10$ s^{-1} [24]. In the absence of experiments that would suggest otherwise, we choose the same K_m and k_{cat} values for all Src-mediated reactions, i.e. also for the phosphorylation of integrin β tails and of PIPKI.

```
PIPKI(S1!1,Y644~U).SRC(SH3!1,Y419~P) -> PIPKI(S1!1,Y644~P).SRC(SH3!1,Y419~P) k8
```

Rule 9: Integrin phosphorylation Integrins have to be in an open conformation, SRC bound, but not necessarily bound by ligand.

```
SRC(SH3!1,Y419~P).INT(BD!1,NPXY~U) -> SRC(SH3!1,Y419~P).INT(BD!1,NPXY~P) k9
```

Rule 10: Tal and PIPKI interaction In the quiescent state most talin cannot bind integrins because of self-inhibitory interactions [25]. The inhibited forms can, however, still bind PIPKI. The affinity between PIPKI and talin has been determined as 170 nM [10, 26]. Since PIPKI exists as a dimer, we model two talin binding sites leading to TAL:PIPKI dimers and trimers. Without loss of generality we assume a sequential binding.

```
TAL(Loc~PM,BD) + PIPKI(Loc~PM,T1,T2) <->
TAL(Loc~PM,BD!1).PIPKI(Loc~PM,T1!1,T2) k10on, k10off
TAL(Loc~PM,BD) + PIPKI(Loc~PM,T1!+,T2) <->
TAL(Loc~PM,BD!1).PIPKI(Loc~PM,T1!+,T2!1) k10on, k10off
TAL(Loc~CP,BD) + PIPKI(Loc~CP,T1,T2) <->
TAL(Loc~CP,BD!1).PIPKI(Loc~CP,T1!1,T2) k10on, k10off
TAL(Loc~CP,BD) + PIPKI(Loc~CP,T1!+,T2) <->
TAL(Loc~CP,BD!1).PIPKI(Loc~CP,T1!+,T2!1) k10on, k10off
```

Rule 11a: Activation of talin Most cellular talin is unable to bind integrins because of inhibitory self-interactions [25]. Talin can be activated by PIPKI which produces PIP2 [27, 28]. PIP2 is highly unstable [29], and its production, decay and diffusion are not modelled explicitly. We rather assume that talin must be bound to membrane-bound, active PIPKI for activation. The rate of PIPKI-dependent PIP2 production and talin activation has not been measured, and we use $k11a = 0.1s^{-1}$.

```
TAL(Loc~PM,Conf~C,BD!1).PIPKI(Loc~PM,T1!1,Y644~P) ->
TAL(Loc~PM,Conf~O,BD!1).PIPKI(Loc~PM,T1!1,Y644~P) k11a
TAL(Loc~PM,Conf~C,BD!1).PIPKI(Loc~PM,T2!1,Y644~P) ->
TAL(Loc~PM,Conf~O,BD!1).PIPKI(Loc~PM,T2!1,Y644~P) k11a
```

Rule 11b: Activation of talin We also assume a PIPKI independent activation of talin at the membrane with a very low rate of $k11b = 10^{-3}$.

```
TAL(Loc~PM,Conf~C) -> TAL(Loc~PM,Conf~O) k11b
```

Rule 12: Inactivation of talin Only if it is unbound

```
TAL(Conf~O,BD) -> TAL(Conf~C,BD) k12
```

Rule 13: Src de-phosphorylation We include constitutive Src, integrin, and PIPKI dephosphorylation at rates 0.1 s^{-1}. These rates have not been measured directly in experiments, but data exist that provide some bounds. Thus in talin knock-out cells Src activity drops to 50 % of its maximal value within 15-20 minutes. For large dephosphorylation rates there is little Src activation and the system rapidly equilibrates. For small dephosphorylation rates rapid Src deactivation is impossible. The rate of Src dephosphorylation must therefore be about $k13 = 0.1s^{-1}$.

```
SRC(Conf~O,Y419~P) -> SRC(Conf~O,Y419~U) k13
```

Rule 14: PIPKI de-phosphorylation Must be unbound by Src

```
PIPKI(Y644~P,S1) -> PIPKI(Y644~U,S1) k14
```

Rule 15: integrin dephosphorylation Must be unbound by Src

```
INT(NPXY~P,BD) -> INT(NPXY~U,BD) k15
```

Rule 16a: talin shuttling

```
TAL(Loc~CP,BD) <-> TAL(Loc~PM,BD) k16on, k16off
```

Rule 16b: PIPKI shuttling incase of dimers

```
TAL(Loc~CP,BD!1).PIPKI(Loc~CP,T1!1,T2,S1) <->
TAL(Loc~PM,BD!1).PIPKI(Loc~PM,T1!1,T2,S1) k16on, k16off
```

Rule 16c: DOK shuttling (same parameters as for talin)

```
DOK(Loc~CP,BD) <-> DOK(Loc~PM,BD) k16on, k16off
```

Rule 17a: PIPKI shuttling Phosphatidylinositol phosphate kinase type Iγ (PIPKI) appear not to bind well to membrane lipids and PIPKI is therefore assumed to be homogeneously distributed between membrane compartment and cytoplasm [30], i.e PIPKI shuttles between membrane and cytoplasm at rate 1 s^{-1} in both directions.

```
PIPKI(Loc~CP,T1,T2,S1) <-> PIPKI(Loc~PM,T1,T2,S1) k16on, k17offA
```

Rule 17b: PIPKI shuttling in case of trimers

```
TAL(Loc~CP,BD!1).PIPKI(Loc~CP,T1!1,T2!2,S1).TAL(Loc~CP,BD!2) <->
TAL(Loc~PM,BD!1).PIPKI(Loc~PM,T1!1,T2!2,S1).TAL(Loc~PM,BD!2) k16on, k17offB
```

Compartment modeling

We consider two reaction compartments: cytoplasm (CP) and plasma membrane (PM). TAL, PIPKI and DOK can shuttle between both compartments. Since the CP volume is about 20 fold larger compared with the effective PM reaction volume, the shuttling rates have to account for the change in species concentration due to shuttling. Therefore, we normalize the compartment exchange fluxes on a per-species basis as detailed below. Note, that this approach differs from the standard SBML formulation, which relies on an extensive description of species levels (i.e. species levels are treated as absolute numbers and not as concentrations) [31].

Let A and B be two compartments with reaction volume V_A and V_B, respectively. Let n_A be the number of particles in compartment A and n_B the number of particles in compartment B. For simplicity we assume a constant number of particles.

$$n_T \quad = n_A + n_B$$

The concentrations in the compartments are denoted as $x_A = n_A/V_A$ and $x_B = n_B/V_B$. We want to describe the change in concentration due to the flux of particles between the compartments. We assume large particle numbers, such that the problem can be formulated in terms of a set of differential equations.

$$\dot{x}_A \quad = -\alpha_{A\to B} + \alpha_{B\to A} \tag{1}$$

$$\dot{x}_B \quad = -\beta_{B\to A} + \beta_{A\to B} \tag{2}$$

We will assume mass-action kinetics, i.e., all fluxes are proportional to the concentrations of the reacting species.

$$\alpha_{A\to B} = a_1 x_A$$
$$\alpha_{B\to A} = a_2 x_B$$
$$\beta_{B\to A} = b_1 x_B$$
$$\beta_{A\to B} = b_2 x_A$$

Due to conservation of particles $\dot{n}_A + \dot{n}_B = 0$, and therefore

$$\dot{x}_A V_A + \dot{x}_B V_B = -a_1 n_A + a_2 \frac{V_A}{V_B} n_B - b_1 n_B + b_2 \frac{V_B}{V_A} n_A \stackrel{!}{=} 0.$$

The last equation can be rearranged to

$$n \cdot v = 0,$$

where $n = (n_A, n_B)$ and $v = (b_2 \frac{V_B}{V_A} - a_1, a_2 \frac{V_A}{V_B} - b_1)^T$. This equation must hold for all n. As an example, consider the two equations $n_1 \cdot v = 0$ and $n_2 \cdot v = 0$ for linearly independent vectors n_1 and n_2, which can only hold true for $v = (0, 0)$. This is therefore the general solution for arbitrary n. It follows

$$\begin{aligned} b_1 &= a_2 \tfrac{V_A}{V_B} \\ b_2 &= a_1 \tfrac{V_A}{V_B}. \end{aligned}$$

Inserting these into the original equation for x_B leads to

$$\dot{x}_B = -(a_1 V_A x_B + a_2 V_A x_A)/V_B.$$

Defining the new parameters $\tilde{a_1} = a_1 V_A$ and $\tilde{a_2} = a_2 V_A$, we can rewrite the original equations in terms of the new parameters as

$$\begin{aligned} \dot{x}_A &= (-\tilde{a_1} x_A + \tilde{a_2} x_B)/V_A & (3) \\ \dot{x}_B &= (-\tilde{a_2} x_B + \tilde{a_1} x_A)/V_B. & (4) \end{aligned}$$

The dimension of $\tilde{a_1}$ and $\tilde{a_2}$ are volume over time and are explicitly referencing V_A. However, we can by the same approach define parameters $\tilde{b_1} = b_1 V_B$ and $\tilde{b_2} = b_2 V_B$ referencing volume V_B and derive the equations in terms of $\tilde{b_1}$ and $\tilde{b_2}$.

$$\begin{aligned} \dot{x}_A &= (-\tilde{b_2} x_A + \tilde{b_1} x_B)/V_A \\ \dot{x}_B &= (-\tilde{b_1} x_B + \tilde{b_2} x_A)/V_B \end{aligned}$$

From this it is apparent that $\tilde{a_1} = \tilde{b_2} \equiv k_{A \to B}$ and $\tilde{a_2} = \tilde{b_1} \equiv k_{B \to A}$.

Particle numbers might not be conserved due to production or decay occurring within compartments. However, in mass-action kinetics velocities are proportional to concentrations: changing the reaction volume while keeping the concentrations fixed, does not change the velocity of a reaction. Therefore, the transformations $a_1 \to \tilde{a_1}$ and $a_2 \to \tilde{a_2}$ do not affect other fluxes within the compartments, whose rates are volume independent. Therefore the general form of the reaction kinetics including production, decay and compartment exchange is:

$$\begin{aligned} \dot{x}_A &= (-\tilde{a_1} x_A + \tilde{a_2} x_B)/V_A + f_{prod}(x_A) - f_{deg}(x_A) & (5) \\ \dot{x}_B &= (-\tilde{a_2} x_B + \tilde{a_1} x_A)/V_B + f_{prod}(x_B) - f_{deg}(x_B). & (6) \end{aligned}$$

Evaluation of sample size

Exhaustive sampling of the biologically feasible parameter range is computationally infeasible. Since most of the parameter combinations lead to qualitatively similar model dynamics we are interested in the effective number of samples needed to get a comprehensive picture of the possible model dynamics. A sample refers to a full set of model parameters (i.e. one parameter vector), were each element is drawn from its respective exponential distribution. To this end we monitor the convergence of the time-dependent mean of the model observables for an increasing number of samples by a blocking procedure. Samples of size $N = \{10, 10^2, 10^3, 10^4, 10^5\}$ are divided into 10 blocks. For each sample size we calculate the overall mean as well as the standard deviation of the means of all 10 blocks. Next, we compute the coefficient of variation (CV), i.e., the standard deviation divided by the mean, for each sample size. This gives us a CV value for each sample size, time point and model observable (Figure S1). The analysis shows, that the average signaling dynamics is sufficiently well captured with 10^5 samples, i.e. $CV < 0.1$ for all observables and time points.

References

1. Vitte J, Benoliel AM, Eymeric P, Bongrand P, Pierres A (2004) Beta-1 integrin-mediated adhesion may be initiated by multiple incomplete bonds, thus accounting for the functional importance of receptor clustering. Biophys J 86: 4059-74.

2. Cox EA, Sastry SK, Huttenlocher A (2001) Integrin-mediated adhesion regulates cell polarity and membrane protrusion through the rho family of gtpases. Molecular Biology of the Cell 12: 265–77.

3. Neff NT, Lowrey C, Decker C, Tovar A, Damsky C, et al. (1982) A monoclonal antibody detaches embryonic skeletal muscle from extracellular matrices. J Cell Biol 95: 654-66.

4. Akiyama SK, Hasegawa E, Hasegawa T, Yamada KM (1985) The interaction of fibronectin fragments with fibroblastic cells. J Biol Chem 260: 13256-60.

5. Wiseman PW, Brown CM, Webb DJ, Hebert B, Johnson NL, et al. (2004) Spatial mapping of integrin interactions and dynamics during cell migration by image correlation microscopy. J Cell Sci 117: 5521-34.

6. Calderwood DA, Yan B, de Pereda JM, Alvarez BG, Fujioka Y, et al. (2002) The phosphotyrosine binding-like domain of talin activates integrins. J Biol Chem 277: 21749–21758.

7. Anthis NJ, Haling JR, Oxley CL, Memo M, Wegener KL, et al. (2009) Beta integrin tyrosine phosphorylation is a conserved mechanism for regulating talin-induced integrin activation. J Biol Chem 284: 36700–36710.

8. Saltel F, Mortier E, Hytonen VP, Jacquier MC, Zimmermann P, et al. (2009) New pi(4,5)p2- and membrane proximal integrin-binding motifs in the talin head control beta3-integrin clustering. J Cell Biol 187: 715–731.

9. Ling K, Doughman RL, Iyer VV, Firestone AJ, Bairstow SF, et al. (2003) Tyrosine phosphorylation of type igamma phosphatidylinositol phosphate kinase by src regulates an integrin-talin switch. J Cell Biol 163: 1339–1349.

10. de Pereda JM, Wegener KL, Santelli E, Bate N, Ginsberg MH, et al. (2005) Structural basis for phosphatidylinositol phosphate kinase type igamma binding to talin at focal adhesions. J Biol Chem 280: 8381–8386.

11. Moser M, Legate KR, Zent R, Fassler R (2009) The tail of integrins, talin, and kindlins. Science 324: 895–899.

12. Hynes RO, Lively JC, McCarty JH, Taverna D, Francis SE, et al. (2002) The diverse roles of integrins and their ligands in angiogenesis. Cold Spring Harb Symp Quant Biol 67: 143-53.

13. Tadokoro S, Shattil SJ, Eto K, Tai V, Liddington RC, et al. (2003) Talin binding to integrin beta tails: a final common step in integrin activation. Science 302: 103-6.

14. Plow EF, Ginsberg MH (1981) Specific and saturable binding of plasma fibronectin to thrombin-stimulated human platelets. J Biol Chem 256: 9477-82.

15. Faull RJ, Ginsberg MH (1995) Dynamic regulation of integrins. Stem Cells 13: 38-46.

16. Iber D, Campbell ID (2006) Integrin activation–the importance of a positive feedback. Bull Math Biol 68: 945-56.

17. Kuo SC, Lauffenburger DA (1993) Relationship between receptor/ligand binding affinity and adhesion strength. Biophys J 65: 2191-200.

18. Moy VT, Jiao Y, Hillmann T, Lehmann H, Sano T (1999) Adhesion energy of receptor-mediated interaction measured by elastic deformation. Biophys J 76: 1632-1638.

19. Bell G (1978) Models for the specific adhesion of cells to cells. Science 200: 618-627.

20. Oxley CL, Anthis NJ, Lowe ED, Vakonakis I, Campbell ID, et al. (2008) An integrin phosphorylation switch: the effect of beta3 integrin tail phosphorylation on dok1 and talin binding. J Biol Chem 283: 5420–5426.

21. Roskoski R Jr (2004) Src protein-tyrosine kinase structure and regulation. Biochem Biophys Res Commun 324: 1155-64.

22. Arias-Salgado EG, Lizano S, Sarkar S, Brugge JS, Ginsberg MH, et al. (2003) Src kinase activation by direct interaction with the integrin beta cytoplasmic domain. Proc Natl Acad Sci U S A 100: 13298–13302.

23. Harrison SC (2003) Variation on an src-like theme. Cell 112: 737-40.

24. Songyang Z, Carraway KL 3rd, Eck MJ, Harrison SC, Feldman RA, et al. (1995) Catalytic specificity of protein-tyrosine kinases is critical for selective signalling. Nature 373: 536-9.

25. Goksoy E, Ma YQ, Wang X, Kong X, Perera D, et al. (2008) Structural basis for the autoinhibition of talin in regulating integrin activation. Mol Cell 31: 124-33.

26. Barsukov IL, Prescot A, Bate N, Patel B, Floyd DN, et al. (2003) Phosphatidylinositol phosphate kinase type 1gamma and beta1-integrin cytoplasmic domain bind to the same region in the talin ferm domain. J Biol Chem 278: 31202-9.

27. Paolo GD, Pellegrini L, Letinic K, Cestra G, Zoncu R, et al. (2002) Recruitment and regulation of phosphatidylinositol phosphate kinase type 1 gamma by the ferm domain of talin. Nature 420: 85–9.

28. Ling K, Doughman RL, Firestone AJ, Bunce MW, Anderson RA (2002) Type i gamma phosphatidylinositol phosphate kinase targets and regulates focal adhesions. Nature 420: 89-93.

29. Hilgemann DW (2007) Local pip(2) signals: when, where, and how? Pflugers Arch 455: 55-67.

30. Giudici ML, Lee K, Lim R, Irvine RF (2006) The intracellular localisation and mobility of type igamma phosphatidylinositol 4p 5-kinase splice variants. FEBS Lett 580: 6933-7.

31. Hucka M, Finney A, Sauro HM, Bolouri H, Doyle JC, et al. (2003) The systems biology markup language (sbml): a medium for representation and exchange of biochemical network models. Bioinformatics 19: 524-31.

Supporting Information Legends

Figure S1. Mean signaling dynamics in dependence of the sample size. Each plot shows the coefficient of variation (standard deviation/mean) in dependance of time (horizontal axis) and sample size (vertical axis) for a selected observable. Mean and standard deviation are calculated by a blocking procedure as described in the text. For a sample size of 10^5 the coefficient of variation is below 0.1 % for all time points and observations.

Supporting Information Figures

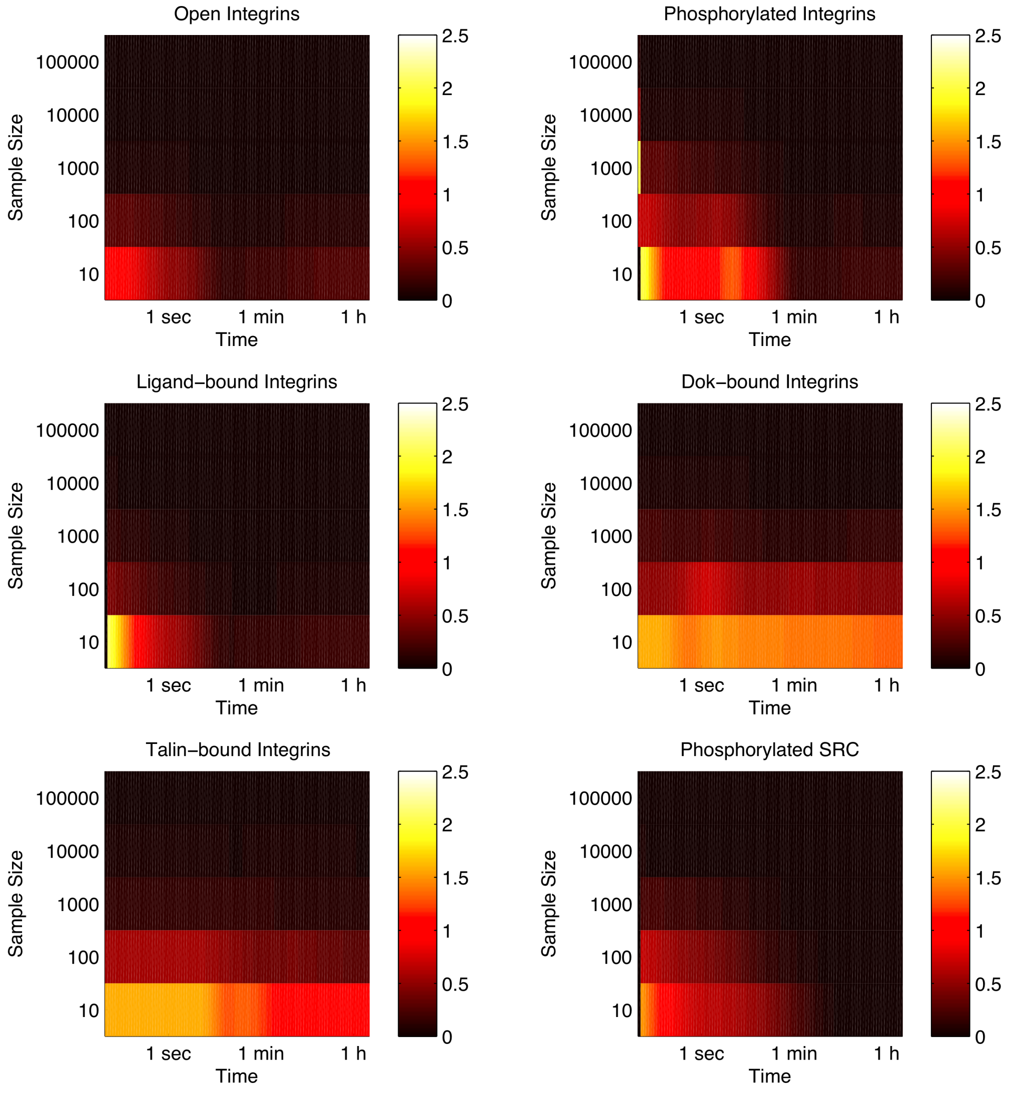

Figure S1
